# Propensity score weighted associations between financial strain and subsequent inflammatory biomarkers of aging among a representative sample of U.S. older adults

**DOI:** 10.1186/s12877-022-03112-5

**Published:** 2022-05-31

**Authors:** Laura J. Samuel, Melissa Hladek, Jing Tian, Laken C. Roberts Lavigne, Sarah E. LaFave, Sarah L. Szanton

**Affiliations:** 1 Johns Hopkins University School of Nursing, 525 North Wolfe St., Baltimore, MD 21205 USA; 2 Department of Biostatistics, Johns Hopkins Bloomberg School of Public Health, Baltimore, MD USA; 3 Department of Health Policy and Management, Johns Hopkins Bloomberg School of Public Health, Baltimore, MD USA

**Keywords:** Socioeconomic factors, Inflammation, Metabolic function, Health equity

## Abstract

**Background:**

Despite known socioeconomic disparities in aging-related outcomes, the underlying physiologic mechanisms are understudied. This study applied propensity score weighting to estimate the effect of financial strain on inflammation-related aging biomarkers among a national sample of older adults.

**Methods:**

Financial strain severe enough to lack money for housing, utilities, medical/prescription bills or food was measured among 4,593 community-dwelling National Health and Aging Trends Study participants aged ≥ 65 years in 2016. Inverse probability propensity score weights were generated based on 2015 background characteristics, including age, gender, race/ethnicity, income to poverty ratio, education, occupation, home ownership, retirement, Sect. 8 housing, Medicaid, food/energy assistance, childhood health, marital status, and U.S. region. Sampling weights additionally accounted for study design and non-response.

**Results:**

In propensity score-weighted analyses adjusting for age, gender, race/ethnicity, 2017 income to poverty ratio and education, those with 2016 financial strain had 15% higher IL-6 (*p* = 0.026) and 20% higher CRP levels (*p* = 0.002) in 2017 than those who were not strained, but did not differ with regard to hemoglobin A1c or CMV. In weighted comparisons, those with financial strain did not differ from those without with regard any 2015 background characteristics.

**Conclusions:**

These results strengthen the etiologic evidence suggesting that financial strain increases inflammatory biomarkers among older adults. Importantly, inflammation is likely a key physiologic pathway contributing to socioeconomic disparities. Therefore, research is needed to address financial strain.

**Supplementary Information:**

The online version contains supplementary material available at 10.1186/s12877-022-03112-5.

## Introduction

About one-third of older adults in the United States experience financial strain [1], or difficulty making ends meet. Financially strained older adults have a higher risk of physical disability, dementia, and earlier mortality [2–4]. However, the underlying mechanisms are poorly understood because financial strain cannot be randomly assigned for an experimental study. One study applying propensity score weighting found that financial strain specific to housing costs was associated with higher risk of poor health, hypertension, and arthritis, suggesting that lack of money for basic needs may cause poor health [5]. However, the specific underlying physiological pathways leading from financial strain to aging-related health outcomes are not well understood.

Inflammation is one physiologic pathway that likely is partly responsible for linking financial strain to aging-related outcomes. It is already known that aging itself is associated with increased production of pro-inflammatory cytokines like interleukin-6 (IL-6) and C-reactive protein (CRP) [6]. This perpetuates a chronic low-grade inflammatory phenotype that makes older adults more vulnerable to metabolic dysfunction [7], including increased hemoglobin A1c levels. Also, inflammation and latent cytomegalovirus (CMV) infection co-occur and may influence each other [8]. These biomarkers—IL-6, CRP, CMV, and hemoglobin A1c—predict earlier disability [9–11], dementia [12–14], and mortality [15–17]. This is likely due to fact that inflammatory biomarkers also predict the cardiometabolic conditions that contribute to risk for disability and dementia and that they are increased by other risk factors, such as smoking [18, 19]. Together, these studies suggest that inflammatory pathways contribute to these aging-related outcomes. However, not all older adults have the same risk for poor outcomes.

There is cross-sectional evidence showing relationships between financial strain and inflammation-related biomarkers. Studies conducted among a convenience sample [20], and a regional sample [21], have shown that financial strain is associated with higher levels of CRP and IL-6 [20, 21]. Among a national sample of U.S. older adults, financial strain was associated with higher hemoglobin A1c [22]. Together, these cross-sectional studies show correlation, but longitudinal studies with stronger causal methods among nationally representative samples are needed to elucidate underlying mechanisms.

Importantly, each of these inflammatory biomarkers capture distinct relationships between stress and health [23]. For example, secretion of CRP and IL-6 are triggered by the physiologic stress response, and may contribute to metabolic dysfunction including elevated hemoglobin A1c [24]. However, elevated hemoglobin A1c is also likely sensitive to environmental factors including food insecurity [25]. CMV may capture distinct relationships between social environmental characteristics and health because of socioeconomic and geographic differences in latent infection [26]. Determining the specific associations between financial strain and each of these individual biomarkers would allow for better understanding of how to mitigate the impact of financial strain on health.

This study sought to elucidate mechanisms linking financial strain with inflammatory biomarkers of aging among a nationally representative sample of U.S. older adults. This study applies a propensity score approach to test the hypothesis that financial strain predicts higher subsequent levels of IL-6, CRP, hemoglobin A1c, and CMV antibodies.

## Methods

### Study design and sample

The National Health and Aging Trends Study (NHATS) recruited a cohort of U.S. Medicare beneficiaries aged 65 years and older using stratified random sampling in 2011 and replenished the sample in 2015 [27]. NHATS study design is described in detail elsewhere [28]. NHATS participants were interviewed at home annually by trained interviewers. Predictors of financial strain were measured in 2015, financial strain was measured in 2016, and outcomes were measured in 2017. In 2017, all self-responding participants (*n* = 5,266) were asked to provide a blood spot specimen and 93% (*n* = 4,903) consented to do so. Of those who consented, 95.7% (*n* = 4,691) were able to provide a blood specimen. Sampling weights from NHATS accounted for study design, attrition between 2015 and 2017, and non-participation in the blood spot portion of the study so that results can be generalized to the U.S. population of community-dwelling adults over age 67 years. Due to potential qualitative differences in financial strain, these analyses excluded 98 residential care participants, leaving an analytic sample of 4,593. Due to a high likelihood of active infection [29], 139 individuals with CRP levels higher than three standard deviations above the mean were also excluded from CRP and IL-6 analyses. NHATS was approved by the Johns Hopkins Bloomberg School of Public Health IRB (#2083) and participants provided informed consent. These analyses were deemed exempt by the local institutional review board because they were determined to not be human subjects research.

### Exposure measures

The exposure of interest in this study was 2016 financial strain. Participants were classified as having financial strain if they experienced strain in any of four domains severe enough so they lacked money to pay the (1) rent/mortgage, (2) utility bills, or (3) medical/prescription bills in the past year or (4) skipped any meals because there was not enough money to buy food in the past month. Since compromise(s) of in these four domains of basic needs may influence health and because of evidence of trade-off decisions over time among low-income older adults [30, 31], we were interested in comparing older adults who experienced any type of financial strain to those with no financial strain. Additional participant characteristics were measured in 2015 including demographic characteristics, socioeconomic characteristics, utilization of public benefits, childhood health, U.S. region, and marital status. Demographic characteristics included age, gender, and race/ethnicity [White (ref.), Black, Hispanic, and other race]. Socioeconomic characteristics included income to poverty ratio, which was calculated as the ratio of household income to the relevant US Census Bureau poverty threshold for individuals aged ≥ 65 years based on household size and year, education [< high school, high school, some college, and ≥ Bachelor’s degree], longest occupation held using the U.S. Census classifications [management/professional occupation (ref.), service, sales/office, construction/farming, production, and homemaker], homeownership [rent (ref.), own with mortgage, own without payments], and retirement status (no/yes). Utilization of public benefits included measures for receipt in the past year of Sect. 8 housing, Medicaid, and food or energy assistance. Childhood health was classified as [excellent (ref.), very good, good, fair, or poor]. Marital status was classified as [married (ref.), separated/divorced, widowed, never married]. U.S. region was based on the Census classification [New England (ref.), Mid-Atlantic, East North Central, West North Central, South Atlantic, East South Central, West South Central, Mountain, Pacific]. To account for time-variant exposures, the 2017 values for income to poverty ratio and diabetes status were also recorded. Since health conditions likely lie on the causal pathway between financial strain and inflammation-related biomarkers, health variables were not included in these analyses.

### Outcome measures

Outcome biomarkers measured in 2017 included interleukin 6 (IL-6) in pg/ml, hemoglobin A1c in %, anti-cytomegalovirus IgG antibodies (CMV) in AU/ml, and high-sensitivity C-reactive protein (CRP) in mg/L. Blood spots were collected on a card, dried, frozen, and shipped to the University of Washington School of Medicine for processing and analyses as described elsewhere [32]. IL-6, CMV, and CRP were measured with sandwich ELISA and hemoglobin A1c was measured with a Variant II Hemoglobin Testing System (Bio-Rad Laboratories, Hercules, CA). Plasma-equivalent values were used in these analyses rather than raw values for dried blood specimens to aid clinical interpretability. Assay values were highly correlated with plasma equivalent values for IL-6 (*r* = 0.93), hemoglobin A1c (*r* = 0.98), CMV (*r* = 0.98), and CRP (*r* = 0.99).

### Statistical analyses

#### Propensity score model

This study employed propensity score weighting to estimate the Average Treatment Effect (ATE) of financial strain on inflammation-related biomarkers using SAS 9.4 software. Since financially strained older adults differ from non-strained older adults with regard to numerous characteristics, propensity score weighting was used to create groups that are balanced with regard to these characteristics [33]. First, a propensity score logistic regression model (proc surveylogistic) used 2016 financial strain as an outcome and included 2015 values for participant characteristics that not only temporally precede but also theoretically predict both financial strain and biomarkers and do not lie on the causal pathway between them [33]. Characteristics included in the propensity score model were age, gender, race/ethnicity, income to poverty ratio, education, occupation, home ownership, retirement status, Sect. 8 housing receipt, Medicaid use, receipt of food or energy assistance, childhood health, marital status, and U.S. Census Region. To account for differential experiences of discrimination among Black vs. White older adults over the lifespan, the propensity score model also included interactions between Black race with childhood health, education, and home ownership. Balance was considered to have been achieved if the standardized mean difference across groups was reduced and there was no statistically significant differences in the weighted sample [33]. After developing a propensity score model that balanced covariates across financial strain groups, results from the propensity score model were used to generate inverse probability weights, which among those with financial strain was 1/propensity score and among those without financial strain was 1/(1-propensity score). To account for extreme values, inverse probability weights were truncated at the 95^th^ percentile [33].

#### Outcome analyses

Due to skew, outcome values were *ln*-transformed prior to testing hypothesized relationships between 2016 financial strain and 2017 biomarkers using linear regression. Model 1 applied only sampling weights provided by NHATS to account for study design and attrition, not propensity-score weights, and adjusted for 2017 diabetes status in the hemoglobin A1c model. Model 2 additionally adjusted for age, gender, race/ethnicity, 2017 income to poverty ratio, and education in all models. Model 3 adjusted for the same covariates as in Model 2 but estimated the average treatment effect by using analytic weights, which had been calculated as the cross-product of the sampling weights and the inverse probability weights generated from the propensity score model. The analytic weights addresses confounding bias by improving balance (i.e. exchangeability) across financial strain groups. Therefore, Model 3 produces doubly robust estimates with regard to demographic and socioeconomic characteristics.

Two sensitivity analyses were conducted. First, we tried alternative weight truncation at the 90^th^ and 99^th^ percentiles. Second, due to skew in propensity score values for those without financial strain, participants with propensity scores > 0.5 were excluded to evaluate influential observations and address potential comparison of non-exchangeable groups.

## Results

About 6% of the sample had severe enough financial strain in 2016 so they lacked money for basic need(s), which translates into about 2,240,825 U.S. older adults (Table 1). Prior to propensity score weighting, participants who experienced financial strain tended to be younger, have a lower average income to poverty ratio, and were less likely to be White, have graduated high school, have had a professional occupation, or own a home. They were also less likely to have had excellent childhood health and more likely to be separated/divorced than married and to have received Sect. 8 housing, Medicaid, and food or energy assistance. Participants did not differ with regard to gender, retirement status, or U.S. region.

**Table 1:** Selected 2015 background characteristics of community-dwelling NHATS participants based on 2016 financial strain status before propensity score weighting (*n* = 4,335)

	**No financial strain**	**Financial strain**	***p* value**	**Standardized mean difference**
**N (%)**	**4,062 (94)**	**273 (6)**	**0.0001**	**-0.31**
Age %			**0.0001**	**-0.31**
65 to 69 (ref.)	**32.1**	**42.9**		
70 to 74	**29.3**	**31.0**		
75 to 79	**19.5**	**15.4**		
80 to 84	**11.3**	**7.9**		
85 to 89	**5.7**	**2.3**		
90 +	**2.0**	**0.5**		
Gender %			0.19	0.09
Male (ref.)	45.2	39.5		
Female	54.8	60.5		
Race/ethnicity %			** < 0.0001**	**0.38**
White (ref.)	**83.3**	**54.0**		
Black	**7.2**	**21.2**		
Hispanic	**6.7**	**16.9**		
Other	**2.8**	**7.9**		
Mean income to poverty ratio mean (SE)	**4.6 (0.2)**	**2.1 (0.3)**	** < 0.0001**	**-0.25**
Educational achievement %			** < 0.0001**	**-0.23**
< High school (ref)	**14.1**	**27.5**		
High school	**25.1**	**25.3**		
Some college	**29.4**	**31.1**		
Bachelors or higher	**31.3**	**16.1**		
Occupation %			** < 0.0001**	**0.16**
Professional (ref.)	**41.6**	**26.1**		
Service	**10.8**	**20.8**		
Sales/office	**20.7**	**20.7**		
Construction/farming	**10.1**	**9.7**		
Production	**14.8**	**21.8**		
Homemaker	**2.0**	**0.9**		
Homeownership %			** < 0.0001**	**-0.69**
Rent (ref.)	**18.6**	**47.3**		
Own with mortgage	**30.3**	**36.6**		
Own without payments	**51.3**	**16.1**		
Retirement status %			0.42	-0.03
No (ref.)	55.7	58.9		
Yes	44.3	41.1		
Section 8 housing %			** < 0.0001**	**0.27**
No (ref.)	**97.0**	**87.8**		
Yes	**3.0**	**12.2**		
Medicaid (%)			** < 0.0001**	**0.30**
No (ref.)	**90.7**	**75.2**		
Yes	**9.3**	**24.8**		
Food or energy assistance %			** < 0.0001**	**0.47**
No (ref.)	**91.7**	**65.7**		
Yes	**8.3**	**34.3**		
Childhood health %			** < 0.0001**	**0.26**
Excellent (ref.)	**52.5**	**38.7**		
Very good	**27.0**	**26.4**		
Good	**14.7**	**22.0**		
Fair	**4.5**	**7.3**		
Poor	**1.3**	**5.5**		
Marital status %			** < 0.0001**	**0.22**
Married (ref.)	**61.1**	**42.2**		
Separated/divorced	**13.6**	**29.4**		
Widowed	**22.0**	**23.9**		
Never married	**3.3**	**4.6**		
U.S. region %			0.19	-0.05
New England (ref.)	5.8	5.7		
Mid-Atlantic	11.8	16.2		
East North Central	13.9	11.7		
West North Central	9.8	11.2		
South Atlantic	20.1	17.7		
East South Central	6.7	6.0		
West South Central	11.0	16.6		
Mountain	3.1	2.6		
Pacific	17.9	12.3		

Sampling weights were applied to all analyses so that inferences can be drawn to 2017 population of US adults aged 67 and older. Boldface indicates statistical significance

*NHATS* National Health and Aging Trends Study

**Fig. 1:**
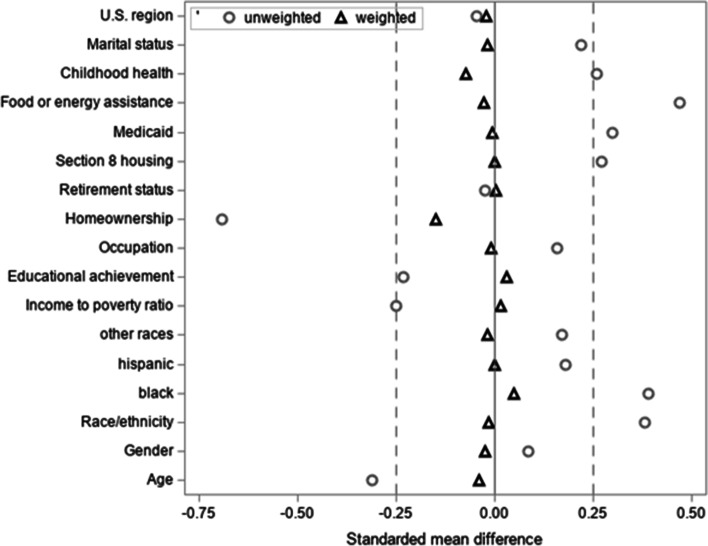
Balance in 2015 background characteristics based on the standardized mean difference between National Health and Aging Trends Study participants with financial strain in 2016 and those without financial strain, comparing estimates obtained before and after applying propensity score weights

The standardized mean difference for 2015 background characteristics was reduced 87% overall after applying propensity score weights, from -0.31 to -0.038, and reduced 60% to 100% for each covariate; absolute standardized difference values ranged from 0.05 to 0.69 prior to weighting and 0 to 0.15 after weighting (Table 2). Improved covariate balance is depicted in Fig. 1. Five financially strained participants with extremely high propensity scores (weights exceeding 4,479,225) were excluded from outcomes analyses in propensity score weighted analyses to avoid off-support inferences. After excluding these individuals, there was considerable overlap in the propensity score distribution comparing financially strained to non-strained participants (Supplemental Fig. 1), suggesting that comparisons across the groups were appropriate.

**Table 2:** Selected propensity score weighted 2015 background characteristics of community-dwelling NHATS participants based on 2016 financial strain status (*n* = 4,335)

	**No financial strain**	**Financial strain**	***p* value**	**Standardized mean difference**
**N (%)**	**4,062 (50)**	**268 (50)**	0.69	-0.038
Age %			0.69	-0.04
65 to 69 (ref.)	33.0	44.9		
70 to 74	29.1	21.3		
75 to 79	19.5	15.0		
80 to 84	11.0	12.7		
85 to 89	5.6	5.0		
90 +	1.8	1.1		
Gender %			0.76	-0.03
Male (ref.)	44.8	48.8		
Female	55.2	51.2		
Race/ethnicity %			0.81	-0.02
White (ref.)	82.1	83.4		
Black	7.8	6.8		
Hispanic	7.2	7.2		
Other	2.9	2.6		
Mean income to poverty ratio mean (SE)	4.4 (0.2)	4.7 (1.5)	0.85	0.02
Educational achievement %			0.71	0.03
< High school (ref)	14.7	16.5		
High school	25.1	23.5		
Some college	29.5	14.7		
Bachelors or higher	30.7	45.3		
Occupation %			0.82	-0.01
Professional (ref.)	41.0	40.4		
Service	11.1	6.9		
Sales/office	20.8	31.4		
Construction/farming	9.9	8.7		
Production	15.3	10.8		
Homemaker	1.9	1.7		
Homeownership %			0.09	-0.15
Rent (ref.)	19.9	20.3		
Own with mortgage	30.5	49.5		
Own without payments	49.6	30.2		
Retirement status %			0.96	0.00
No (ref.)	56.1	55.5		
Yes	43.9	44.5		
Section 8 housing %			0.99	0.00
No (ref.)	96.4	96.4		
Yes	3.6	3.6		
Medicaid (%)			0.93	-0.00
No (ref.)	89.9	90.1		
Yes	10.1	9.9		
Food or energy assistance %			0.67	-0.03
No (ref.)	90.2	91.5		
Yes	9.8	8.5		
Childhood health %			0.43	-0.07
Excellent (ref.)	52.3	62.8		
Very good	26.9	20.3		
Good	14.9	11.9		
Fair	4.5	4.1		
Poor	1.3	0.8		
Marital status %			0.91	-0.02
Married (ref.)	60.0	64.8		
Separated/divorced	14.5	8.9		
Widowed	22.2	21.2		
Never married	3.3	5.1		
U.S. region %			0.76	-0.02
New England (ref.)	5.9	2.1		
Mid-Atlantic	11.5	23.4		
East North Central	13.9	7.3		
West North Central	10.0	9.9		
South Atlantic	20.2	17.6		
East South Central	6.8	15.5		
West South Central	11.4	7.2		
Mountain	3.2	4.9		
Pacific	17.0	12.2		

In addition to propensity score weights, sampling weights were applied to all analyses so that inferences can be drawn to 2017 population of US adults aged 67 and older. Boldface indicates statistical significance

*NHATS* National Health and Aging Trends Study

In unadjusted models (Model 1, Table 3), financially strained older adults were estimated to have 22% higher IL-6 levels, 26% higher CMV antibody titers, and 28% higher CRP levels. Adjusting for diabetes diagnosis, they had 3% higher hemoglobin A1c levels. After additionally adjusting for age, gender, race/ethnicity, income to poverty ratio, and education (Model 2, Table 3), they had 18% higher expected IL-6 and 27% higher CRP, but did not differ with regard to hemoglobin A1c level or CMV antibodies.

**Table 3:** Associations between 2016 financial strain with 2017 inflammatory biomarkers of aging among NHATS participants

	**Estimate (SE), *p*-value**		
Outcomes, sample size	Model 1^a^	Model 2^b^	Model 3^c^
IL-6 (pg/ml) (*n* = 3,762)	**0.22 (0.08), 0.008**	**0.18 (0.08), 0.028**	**0.15 (0.07), 0.026**
Hemoglobin A1c (%) (*n* = 4,106)	**0.03 (0.01), 0.015**	0.01 (0.01), 0.510	0.01 (0.01), 0.283
CMV (AU/ml) (*n* = 4,123)	**0.26 (0.08), 0.002**	0.11 (0.09), 0.200	0.10 (0.06), 0.115
CRP (mg/L) (*n* = 3,915)	**0.28 (0.07), < 0.001**	**0.27 (0.08), < 0.001**	**0.20 (0.06), 0.002**

Obtained from linear regression of ln-transformed outcome values. Sample weights were applied to all models so inferences can be drawn to U.S. older adult Medicare beneficiaries. Boldface indicates statistical significance

^a^Adjusted for 2017 diabetes diagnosis status in the hemoglobin A1c model

^b^Additionally adjusted for age, gender, race/ethnicity, 2017 income to poverty ratio, and education

^c^Applied both sampling and propensity score weights to obtain doubly robust estimates by accounting for 2015 background characteristics

*NHATS* National Health and Aging Trends Study

In propensity score weighted analyses, those with financial strain did not differ from non-strained older adults with regard to any measured background characteristics (Table 2). In adjusted models that apply sampling and propensity score weights, those with financial strain had 15% higher typical IL-6 and 20% higher typical CRP levels than those without financial strain, and did not differ with regard to hemoglobin A1c or CMV antibodies (Model 3, Table 3). Sensitivity analyses were performed as described in the methods section and inferences remained unchanged in those models.

## Discussion

Financial strain predicted subsequent IL-6 and CRP levels using a propensity-score approach among a nationally representative sample of U.S. older adults. These results build on those from prior studies reviewed earlier linking financial strain with inflammatory biomarkers [20–22] by providing relatively stronger evidence of an underlying causal relationship. Together with results elsewhere linking inflammatory cytokines to disability, dementia, and mortality in older adults, these results suggest that inflammatory cytokines may account for the disparities in these outcomes based on financial strain exposure.

These results are consistent with results from natural experimental studies that intervened to address low income, which contributes to financial strain. Although natural experimental studies have found evidence of increased smoking and drinking after receiving relatively large sums of money such as lottery winnings or annual casino profits [34, 35], there is also evidence of improvement in other health outcomes after receiving either larger or smaller sums of money, including improvements in IL-6, CRP,[21] cognitive function, heart rate, blood pressure levels [36], obesity, diabetes [37], and mental health [38]. Together, these results suggest that although improved access to socioeconomic resources may worsen some health behaviors, they often improve numerous other health outcomes related to stress, metabolism, and well-being. Additional work is needed to develop and test the health impact of policies and programs aimed at improving socioeconomic resources.

There are possible physiologic explanations for these results. Financial strain experienced chronically over the life course likely repeatedly activates stress response mechanisms, including triggering cortisol secretion in the hypothalamic pituitary adrenal axis, which, in turn, inhibits immune response and triggers IL-6 and CRP secretion [23, 39]. These results are important because there has been much attention given to the harmful impact of early life exposure to stressful experiences, which is a sensitive period [23]. However, results from this study suggest that stressful events may also provoke immune responses among older adults, and this could explain the accumulation of disease and disability burden among socioeconomically disadvantaged older adults and the widening disparities documented over the adult lifespan comparing rich and poor [40]. Importantly, evidence of the ongoing accumulation of physiologic wear and tear during older adulthood suggest that it is not too late to address financial strain in late life. Interventions to attenuate financial strain among older adults may prevent health declines.

Notably, financial strain did not predict levels of CMV antibodies or hemoglobin A1c in adjusted analyses, despite evidence of an association with hemoglobin A1c found in another study [22] and evidence that older adults compromise basic necessities including food when experiencing financial challenges [30, 31, 41]. There are several potential reasons for this. First, it is possible that financial strain does not directly influence hemoglobin A1c or CMV infection. As examples, financial strain tends to co-occur with food insecurity and cost-related medication non-adherence [42] and these other social determinants of health may influence hemoglobin A1c. Second, low income households have been shown to have increased risk of hypoglycemia at the end of the month when food budgets tend to run low [43] but hemoglobin A1c may not be sensitive to temporal glucose fluctuations because it measures average blood sugar over three months. However, it also possible that the mechanisms linking financial strain to hemoglobin A1c and CMV infection occur earlier in the lifespan than older age because CMV infection tends to occur in early life [26] and diabetes tends to occur in mid-life [44]. Future studies in younger populations and using repeated outcome measures are needed to investigate causal mechanisms further. Future studies should consider using methods to strengthen causal inference, such as propensity score approaches.

### Limitations

Although this study was strengthened by accounting for multiple factors which likely capture multiple confounding pathways across the life-course, including childhood health, educational achievement, lifetime occupation, and a large set of late-life exposures, older adults likely have accumulated a large set of exposures earlier in their life which may predict both financial strain and inflammation-related biomarkers but are difficulty to quantify and this study had limited life-course measures. Another limitation is lack of baseline measurement for inflammation-related biomarkers. This study was not able to examine specific indicators of financial strain to estimate their effects on health outcomes. Strengths of this study include the inclusion of a nationally representative sample of U.S. older adults and temporal ordering of exposure and outcome.

### Implications

These results have important clinical, public health, and policy implications. Growing attention to the need to screen for social determinants of health in clinical practice and public health surveillance has led to new guidance. The CMS Accountable Health Communities Health-Related Social Needs Screening Tool [45] can identify financial strain and other social determinants of health in clinical settings. Increased screening for financial strain is important because financial strain is modifiable. Because financial strain is the balance between income and need, it is possible to impact this strain by increasing resources or decreasing expenses. As examples of potential policy solutions to increase resources among older Americans, monthly benefit amounts for Social Security and Supplemental Security Income should be updated to account for the fact that the official poverty threshold has not kept pace with cost of living changes over time [46]. Also, many older adults are not able to utilize public benefits such as the Supplemental Nutrition Assistance Program and affordable housing options because of cumbersome enrollment processes and long wait lists, despite good evidence that participation in the programs is related to better self-rated health, less distress and less health care utilization [47–52]. Efforts to streamline access to these programs may reduce financial strain. Also, lowering prescription drug prices or improving access to generic medications could lower expenses for older people. Each of these efforts may impact subsequent biomarkers and future health.

## Conclusion

This study applied a propensity-score approach to compare financially strained and non-strained older adults. Financially strained older adults had higher levels of subsequent IL-6 and CRP compared with non-strained older adults, but not hemoglobin A1c or CMV antibodies. These results build on those of prior studies by providing stronger evidence that financial strain may provoke an inflammatory response. Together with results from other studies, these results suggest that inflammatory pathways may partly explain socioeconomic disparities in aging-related health outcomes.

## Supplementary Information

**Additional file 1: Figure S1.** Propensity score frequency distributions showing overlap comparing National Health and Aging Trends Study participants with financial strain in 2016 to those without.

## Data Availability

The NHATS data analyzed in the current study are available for research purposes at www.nhats.org.

## Abbreviations

U.S.: United States
NHATS: National Health and Aging Trends Study
IL-6: Interleukin-6
CRP: C-reactive protein
CMV: Cytomegalovirus
