# Supplementary material for: Propensity score weighted associations between financial strain and subsequent inflammatory biomarkers of aging among a representative sample of U.S. older adults
Source: BMC Geriatr. 2022 May 31;22:467. doi: 10.1186/s12877-022-03112-5 (PMC9158352; doi:10.1186/s12877-022-03112-5)
Supplement: Supplementary file 1 — Additional file 1: Figure S1. Propensity score frequency distributions showing overlap comparing National Health and Aging Trends Study participants with financial strain in 2016 to those without. [file 12877_2022_3112_MOESM1_ESM.docx]

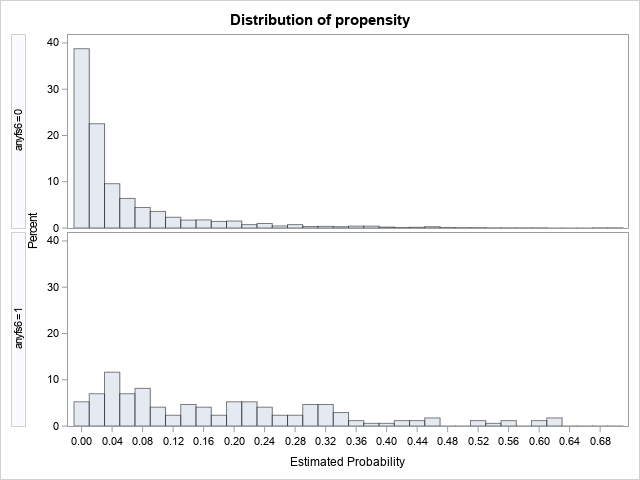


Supplemental Figure 1

Propensity score frequency distributions showing overlap comparing National Health and Aging Trends Study participants with financial strain in 2016 to those without
